# Boo-boos as the building blocks of pain expression: An observational examination of parental responses to everyday pain in toddlers

**DOI:** 10.1080/24740527.2018.1442677

**Published:** 2018-04-12

**Authors:** Melanie Noel, Christine T. Chambers, Jennifer A. Parker, Kate Aubrey, Perri R. Tutelman, Barbara Morrongiello, Chris Moore, Patrick J. McGrath, Natalie L. Yanchar, Carl L. Von Baeyer

**Affiliations:** a Department of Psychology, The University of Calgary and Alberta Children’s Hospital Research Institute, Calgary, AB, Canada; b Department of Psychology and Neuroscience, Dalhousie University, Halifax, NS, Canada; c Centre for Pediatric Pain Research, IWK Health Centre; Halifax, NS, Canada; d Department of Pediatrics, Dalhousie University; IWK Health Centre, Halifax, NS, Canada; e Department of Psychology, University of Guelph, Guelph, ON, Canada; f Departments of Psychiatry and Community Health and Epidemiology, Dalhousie University, Halifax, NS, Canada; g Faculty of Medicine, Centre for Research in Family Health, IWK Health Centre, Halifax, NS, Canada; h Department of Surgery, University of Calgary, Calgary, AB, Canada; i Departments of Clinical Health Psychology and Pediatrics and Child Health, University of Manitoba, Winnipeg, MB, Canada

**Keywords:** everyday pain, acute pain, distress, toddlers, parents

## Abstract

**Background:**

Everyday pain experiences (minor bumps/scrapes) are common in early childhood and create frequent opportunities for socialization of pain behaviors. Nevertheless, everyday pain during the formative toddler period has been largely overlooked.

**Aims:**

The aim of the current study was to describe the frequency and nature of toddlers’ everyday pain experiences, child and parent responses, and the relationship between child characteristics (age, sex, temperament) and responses.

**Methods:**

Fifty-two children aged 12–32 months and their parents were observed at an indoor play facility. Using an observational checklist, trained observers recorded children’s everyday pain incidents and associated child and parent responses.

**Results:**

Overall, 101 pain incidents were observed, the majority of which evoked low levels of pain and distress, which resolved after 1 min. Pain incidents occurred at a rate of 1.02 incidents/child/hour, with 81% of children experiencing at least one incident, which is higher than previous research with preschoolers and daycare staff. Common parent responses included a range of verbal (reassurance) and nonverbal (staying closer, hugging/kissing child) behaviors. Boys were more likely to not exhibit any protective behaviors. Parents were more likely to pick up older toddlers.

**Conclusions:**

Future research should examine the link between self-reported and observed parent responses to child pain in everyday and clinical contexts.

## RÉSUMÉ

**Contexte**: Les expériences douloureuses quotidiennes (bosses et éraflures mineures), courantes chez les jeunes enfants, créent des occasions fréquentes de socialisation des comportements liés à la douleur. Néanmoins, la douleur de tous les jours pendant la période formative des tout-petits a été largement néligée.

**Buts**: Le but de cette étude était de décrire la fréquence et la nature des expériences douloureuses de tous les jours chez les tout-petits, les réponses des enfants et des parents à ces expériences, ainsi que le lien entre les caractéristiques de l’enfant (âge, sexe, tempérament) et ces réponses.

**Méthodes**: Cinquante-deux enfants âgés de 12 à 32 mois et leurs parents ont été observés dans un espace de jeu intérieur. À l’aide d’une grille d’observation, des observateurs dûment formés ont noté les incidents douloureux de tous les jours vécus par les enfants ainsi que les réponses des enfants et des parents à ces incidents.

**Résultats**: Dans l’ensemble, 101 incidents douloureux ont été observés, dont la majorité a suscité des niveaux de douleur et de détresse faibles, résolus après une minute. Les incidents douloureux sont survenus à un rythme de 1,02 incident/enfant/heure et 81 % des enfants ont vécu au moins un incident, ce qui est plus élevé que les études antérieures portant sur des enfants d’âge préscolaire et le personnel de services de garde. Les réponses les plus courantes des parents comprenaient toute une gamme de comportements verbaux (rassurer) et non-verbaux (rester plus proche, étreindre ou embrasser l’enfant). Les garçons étaient plus suceptibles de ne pas démontrer de comportements de protection. Les parents étaient plus susceptibles de prendre les tout-petits plus âgés dans leurs bras.

**Conclusions**: D’autres études devraient se pencher sur le lien entre les réponses auto-déclarées et les réponses observées des parents à la douleur de tous les jours chez les enfants ainsi qu’à la douleur dans un contaxte clinique.

## Introduction

The most common painful incidents are everyday pain experiences, defined as minor bumps and scrapes incurred during everyday activities.^1,2^ These pain experiences are experienced on a frequent basis, particularly early in development (i.e., from the time that children begin walking).^1,2^ Although everyday pain rarely results in serious physical injury, it may provide salient and frequent opportunities for social learning about pain during a formative developmental period. Nevertheless, everyday pain in the toddler years has been largely overlooked and little is known about which responses to these pain experiences are optimal.

To date, only three studies have directly examined everyday pain in children aged 2–7 years.^1–3^ In these studies, observers were trained to use an observational checklist to record the behavioral responses of both children and adult caregivers (i.e., daycare staff). Children experienced more than 0.3 incidents per hour (i.e., 1 incident/3 h), and those experiencing a higher rate of everyday pain responded with longer and more severe distress reactions.^1^ Girls responded with more distress and received more physical comfort from adult caregivers than boys.^1^ Interventions by daycare staff were strongly associated with children’s facial expressions of distress,^2^ although not with trained observers’ ratings of incident severity. This suggests that individual child characteristics that influence their emotional and behavioral responses (e.g., temperament, sex) may be important predictors of adult responses to their everyday pain.

It is likely that parent–child interactions around everyday pain in the toddler period exert a unique and strong influence on the development of young children’s pain expressions and coping. The influence of parental responses to child pain and distress in the context of painful medical procedures and chronic pain is well established.^4,5^ Among infant, child, and adolescent samples, parent behaviors that direct attention toward pain (e.g., verbal reassurance) or that reinforce pain behaviors (e.g., protectiveness) have been linked to increased child pain, distress, and disability^6–13^ and have been conceptualized within an operant learning framework.^4,14,15^ However, the relative influence and function of parent behaviors may differ across stages of child development, and extant research has been largely limited to lab and clinic settings. It is likely that parent responses to frequently occurring pain in children’s typical, naturalistic environments could provide valuable insights into how young children’s pain experiences and pain behaviors develop. Moreover, children’s learning about everyday pain events might be shaped by early learning experiences about risky environments (classical conditioning) as well as social learning about threat from caregivers.

Despite the potential importance of developmental context for socialization of child pain behaviors, research has not examined everyday pain in children under 3 years of age. Furthermore, research has not examined the socializing role of parent responses to everyday pain in young children, despite knowledge that children seek out, and parents provide, emotional information about a range of potentially dangerous and fear-inducing stimuli from the first year of life.^16^ The aim of the current study was to describe the frequency and nature of toddlers’ everyday pain, child and parent responses to those pain experiences, and the relationships between child characteristics (age, sex, temperament) and responses. It was hypothesized that everyday pain experiences, and parental responses to this pain, would occur with high frequency. Moreover, given that perceived threat of pain in younger children might be highest, girls develop greater problems with pain,^17^ and more difficult temperaments have been linked to greater distress during clinical pain,^18^ it was hypothesized that younger age, female sex, and difficult temperament would be related to pain responses (i.e., more protective parent responses, greater pain and distress).

## Method

### Participants

The convenience sample included 52 children (28 boys, 24 girls) and one of their parents (48 mothers, 4 fathers) recruited from the community using advertisements to attend one of three “play parties” (*n* = 17, 17, 18, respectively). Children were included if they spoke English as a first language, were generally healthy (e.g., no chronic illness or recurrent/chronic pain), and were walking by the time of study enrollment, which was operationally defined as the ability to walk across a room unassisted, even if the child is unsteady and falls occasionally.^19^ Children ranged in age from 12 to 32 months (M = 21.04 months; SD = 6.02 months) and parents were between 19 to 47 years (M_age_ = 33.40 years; SD = 4.67 years). The majority (88.5%) of parents were married. All families had two parents living within the home with an average of 1.65 children (SD = 0.68). Most parents identified themselves and their children as Euro-Canadian (88.5% and 86.5%, respectively). Using the Hollingshead Index,^20^ families were best characterized as upper-middle to upper socioeconomic status (M = 25.28; SD = 7.67; class 2). The institutional Research Ethics Board approved this study.

### Measures

#### Demographics form

The demographics form included items to assess parent age, race/ethnicity, relationship to child, number of children living in the home, and socioeconomic status. It also included information on child age, sex, and race/ethnicity.

#### Toddler temperament

The Toddler Temperament Scale (TTS)^21^ assesses temperament in toddlers between the ages of 12 and 36 months and has shown evidence of good reliability and validity.^21^ Parents rate 97 questions about their child’s recent and current behaviors on a six-point Likert scale ranging from 1 (*almost never*) to 6 (*almost always*) based on their own perceptions and observations of their child. Items assess nine dimensions of temperament: activity (e.g., the amount of physical motion during their daily routine); rhythmicity (e.g., regularity of bodily functioning in sleep, hunger, bowel movements, etc.); approach (e.g., their response to new persons, places, events); adaptability (e.g., the ease/difficulty with which their child changes to socially acceptable behavior); intensity (e.g., the amount of energy in response, whether positive or negative); mood (e.g., a general amount of pleasant or unpleasant feelings); persistence (e.g., attention span, how long their child stays with a task or activity); distractibility (e.g., the effect of external stimuli on their ongoing behavior); and threshold (e.g., their general sensitivity or insensitivity to stimuli). Using these subscale scores, children can be categorized as having easy, difficult, or slow-to-warm temperaments. Categorizations were based on the clinical scoring method developed by Fullard and colleagues.^21^ The primary temperament variable used in the current study was the difficult temperament composite score that is calculated as the sum of the Rhythmicity, Approach, Adaptability, Intensity, and Mood subscales.^21,22^ We opted to use the composite score to assess difficulty of temperament because it allowed a continuous score for each child to reflect the *relative* degree of difficultness versus forcing temperament types into discrete categories, which limited variability. Indeed, there was not a lot of variability in the categorizations of each temperament type.

#### Children’s everyday pain and associated child and parent responses

The Dalhousie Everyday Pain Scale–Revised (DEPS-R) was developed for this study based on the Dalhousie Everyday Pain Scale (DEPS),^1^ which is an observational checklist created for the systematic observation of everyday pain incidents in young children. An everyday pain incident was operationally defined as any event of bodily contact with a person or object (including floors, playground equipment) that meets one or both of the following criteria: (1) an observer judges that if she or he had experienced the event in the child’s place, she or he would have felt at least momentary, minor discomfort, or (2) the event results in distress, anger, or protective reactions on the part of the child.^1^ The DEPS has good overall interrater reliability when used with 3- to 7-year-old children (i.e., many items had reliability values in the 0.8–0.9 range).^1,2^

The DEPS was revised for this study because the original scale includes a limited selection of adult responses and does not permit coding of children’s distress both before and after parental response. The basic structure of the DEPS was maintained; however, there were several key changes. First, the adult response section that indicates whether adults engage in distraction, verbal comfort, and physical comfort was replaced with a parent response checklist, which encompassed more varied response options. This more detailed parental response checklist was adapted from a previous unpublished study^23^ that examined 200 parents of school-aged children who rated how often they engaged in various behaviors when their young children had everyday pain. The parent behaviors that were included on the DEPS-R were those that were most frequently endorsed in this previous study, excluding behaviors that could not be exhibited in an active play setting (e.g., “send child to his/her room”). Second, to adapt the DEPS to a younger age group and to facilitate administration, several items were changed: (1) the number of participants involved in the incident and aggressive behaviors was removed because toddlers usually engage in solitary as opposed to group play^24^; (2) variables measuring behavior durations were removed; and (3) a body location diagram was included so that observers could indicate the location of the injured area. Lastly, similar to previous research with children aged 3–5 years,^2^ the Faces Pain Scale–Revised (FPS-R)^25^ was added as an observational tool to code children’s facial expressions of distress immediately following pain incidents and again after 1 min. This was done to allow sufficient time for parents to respond. The FPS-R consists of six gender-neutral faces depicting no pain to the most pain possible. The faces are scored 0, 2, 4, 6, 8, and 10.

The DEPS-R (see Appendix A), like the original DEPS, is divided into the following sections:

1. Behavioral Context. The behavioral context of the injury is rated on two contextual factors, activity level on a scale from 1 (*low*) to 5 (*high*) and tone on a scale from 1 (*calm*) to 5 (*agitated*).
2. Description of Incident. The incident is described in terms of circling the injured body part(s) on a diagram, determining who or what caused the incident (i.e., self, other child, adult, object), and rating the severity of the incident on a scale from 0 (*no hurt*) to 4 (*severe hurt*).
3. Child’s Response. Includes measures of the intensity of child distress immediately post pain incident on a scale from 0 (*none*) to 5 (*high*), intensity of child anger from 0 (*none*) to 5 (*high*), and child facial expression of distress immediately after the pain incident on the FPS-R from 0 (*none*) to 10 (*high*). This section also includes the nature of any protective behaviors (i.e., none, holding the injury, favoring the injury, reduction of activity) as well as the child’s social response (i.e., withdrawal, neutral, help-seeking), facial expression of distress 1 min post pain incident on the FPS-R, and intensity of distress 1 min post pain incident on a scale from 0 (*none*) to 5 (*high*).
4. Parent Response Checklist. This is a 20-item checklist of possible parental responses divided into 10 verbal (e.g., “reassures child that s/he will be OK”; “talks about something else”) and 10 nonverbal (e.g., “rubs the hurt area better”; “hugs or kisses child”) response categories. An additional nonverbal response category (“picking up the child”) was added following pilot testing because it was noted frequently by observers but was not on the original checklist.

### Procedure

Observers were trained on how to use the DEPS-R during two 1-h training sessions, during which they reviewed operational definitions for all scale items. A 2-h pilot play session (*n* = 22) was also held to familiarize observers with the measure and to test the feasibility of the revised checklist in an observational setting.

Parents were recruited from the community, screened for inclusion, and invited to attend a play party with their children, which was held in a spacious indoor play center designed for children aged 6 months to 6 years. The play parties were held approximately 1 month following study enrollment; the play center was reserved exclusively for the present study. The center was divided into two large play rooms and contained engaging equipment to encourage active play, such as a large tree house, climbing wall, rope ladder, swinging bridge, crawl tube, and spiral slide. In addition, there were smaller play structures and a variety of active toys (e.g., balls, skipping ropes) available for use. Safety of the equipment was increased through the use of mats and parental/staff supervision of children.

Prior to the play party, interested parents were mailed a package containing an invitation to the party, the consent form, the demographic questionnaire, and the TTS. A researcher then contacted parents by telephone to discuss the study objectives and answer their questions. To minimize potential reactive effects, parents were told that the purpose of the study was to observe the influence of child temperament on play behaviors. When parents and children arrived at the play party, a researcher greeted them and collected the completed questionnaires and signed consent forms. Parents were asked to place numbered stickers on themselves and their child (on both the front and back of their bodies) so that observers could easily identify parent–child dyads. Parents were simply instructed to interact with their children as they normally would during play.

Observers were trained graduate and undergraduate psychology students who were familiar with the research objectives. Observers were paired (three pairs of two observers each) to obtain interrater reliability estimates and each pair was assigned a different area of the play center to observe (resulting in all of the area being collectively tracked by observers). Observers immediately completed the DEPS-R each time an everyday pain incident was noticed and also conducted follow-up observations 1 min after each incident, which was timed using a stopwatch. Play parties were videotaped; however, the sound and video quality were not acceptable for coding purposes. Before leaving the play party, parents were given a voucher for a complimentary return visit to the play center (value of $7.00 CAD/per child) and children received a “Junior Scientist” certificate.

Following the play party, parents were mailed a debriefing letter that fully described all of the study objectives and the rationale behind not fully disclosing all study objectives at the outset. The letter also provided parents with the option to discard their data if they wished. A researcher subsequently contacted parents via telephone to ensure that the debriefing letter was received and understood and to determine parents’ wishes regarding discarding data. No parent opted to discard their data after being debriefed.

### Data analysis

Interrater reliability was calculated across raters using percent agreement. Frequencies of child pain behaviors as well as parental responses to children’s first everyday pain incidents were calculated. The relationships between children’s age, sex, and difficulty of temperament and types of everyday pain incidents as well as parent responses were examined using correlations and chi-square analyses. Binary logistic regressions were conducted to examine the relationships between child age, sex, and difficulty of temperament and parents’ verbal and nonverbal responses.

## Results

### Interrater agreement

Overall, trained observers recorded 101 everyday pain incidents. Of those, 44 were recorded by one observer and 57 were recorded by two observers. The fact that some observations were recorded by one observer and others by two observers was incidental and due to the busy nature of the play setting; observers were assigned to specific areas and not to individual children and parents. All incidents (101) that were observed by at least one observer were included in the analyses. The 57 incidents recorded by two observers were included in the interrater reliability analyses. Interrater reliability on the DEPS-R items was analyzed using Cohen’s kappa and percent agreement (i.e., total number of concordant observations divided by the total number of paired observations). Percent agreement is reported here and considered appropriate given the low prevalence of many behaviors. Indeed, when items have low prevalence rates, occasional disagreements can cause kappa values to fall drastically and be misleading.^26,27^ Although applying a single standard across variables with different numbers of response options has limitations, for consistency, percent agreement for various codes was classified as follows: 90%–100% excellent, 80%–89% good, 70%–79% fair, and below 70% poor.^28^ See Appendix A for DEPS-R items and possible response options.

Following these guidelines, only variables with an interrater reliability value above 70% were selected for further analysis.^29^ As shown in Table 1, percent agreement for the nominal and parent response variables on the DEPS-R ranged from good to excellent. Similar to previous research with preschoolers,^2^ a small number of variables (i.e., activity level, tone, body location, intensity of anger, and intensity of distress post pain incident) were in the poor range and thus were excluded from analyses.

**Table 1.** Interrater agreement and descriptive statistics for the DEPS-R (*n* = 57).

DEPS-R item (range)	% Agreement	M (SD)
Behavioral context		
Activity level (1–5)^a^	45	—
Tone (1–5)^a^	55	—
Description of incident		
Body location^a^ (three options; upper, lower, both)	34	n/a
Hurt caused by (four options)	80	n/a
Severity of hurt (0–4)^a^	59	—
Child’s response		
Child distress post incident (0–5)^a^	63	—
Child facial distress post incident (0–5)	70	0.91 (1.35)
Intensity of anger (0–5)	89	0.18 (0.63)
Protective behaviors (three options)	93	n/a
Social response (three options)	82	n/a
Child distress 1 min post incident (0–5)	100	0.05 (0.38)
Child facial distress 1 min post incident (0–5)	98	0.07 (0.53)

^a^Variable not included in analyses due to low reliability (<70%).

DEPS-R = Dalhousie Everyday Pain Scale–Revised.

### Frequency and nature of everyday pain incidents

Frequencies of parental responses to the first everyday pain incident are shown in Table 2. During the three 2-h play parties, 81% of children (*n *= 42; 25 boys, 17 girls) experienced at least one everyday pain incident and children collectively experienced a total of 101 incidents. These everyday pain incidents occurred over 99.5 observation hours (adjusted for families who left early), yielding an incident rate of 1.02 incidents per child per hour. During the play parties, children experienced a mean of 1.92 incidents (range = 0–7; SD = 1.67) with 27% (*n* = 14) experiencing only one incident, 25% (*n* = 13) experiencing two incidents, 15% (*n* = 8) experiencing three incidents, 6% (*n* = 3) experiencing four incidents, 4% (*n* = 2) experiencing five incidents, 0% (*n* = 0) experiencing six incidents, and 4% (*n *= 2) experiencing seven incidents.

**Table 2.** Frequency of parental responses to first everyday pain incident.^a^

Parental response	No. (%) of responses (*n* = 42)
Does not notice incident	6 (14.3)
Verbal responses:	
Reassures child that (s)he will be OK	2 (4.7)
Apologizes to child	0 (0)
Sympathizes with child	0 (0)
Encourages child to to something else	0 (0)
Talks about something else	1 (2.4)
Tells child to stop crying	0 (0)
Tells child to be more careful	1 (2.4)
Checks in with child (e.g., “are you OK?”)	9 (21.4)
Gets upsets with, scolds, or threatens child	0 (0)
Other (e.g., says “oops”/“whoops”/“oh-oh”)	9 (21.4)
Nonverbal responses:	
Witnessed incident but shows no reaction	6 (14.3)
Rubs hurt area	2 (4.8)
Hugs/kisses the child	5 (11.9)
Kisses the “booboo” better	1 (2.4)
Stays closer to child	8 (19.0)
Distracts child with a toy or object	0 (0)
Distracts child by pointing to something	0 (0)
Takes child out of play setting	7 (16.7)
Other/picks up child^b^	14 (33.3)

^a^Response types were observed to occur at least once during entire observational period.

^b^This variable was created post hoc and thus reliability could not be calculated.

The majority of everyday pain incidents were inadvertently caused by children themselves (57.4%; e.g., falling while running) or were caused by impact with an object (34.7%; e.g., tripping on ladder), whereas fewer incidents were caused by another child (5.9%; e.g., being hit) or by an adult (2%; e.g., letting go of child). In terms of the severity of the incidents, most incidents were rated by observers as evoking no hurt (18.8% rated as a 0/4) or low hurt (50.5% rated as 1/4 and 24.8% rated as 2/4; e.g., falling lightly on mat) and only a few were rated as more severe (4.0% rated as 3/4 and 2.0% rated as 4/4; e.g., falling off the jungle gym). Most facial expressions immediately after the pain incident (i.e., before the parent intervened) were rated as indicating no evidence (51.5%) of distress, whereas 20.8% had a score of 2, 7.9% had a score of 4 and 6 each, 2.0% had a score of 8, and 5.0% had a score of 10/10. Five percent of FPS-R scores were missing due to the observer not being able to see the child’s face to properly code. Following 76.2% of incidents, no protective behaviors were observed; however, in 11.9% of incidents, children reduced their subsequent activity, in 6.9% they favored the injured body part, and in 4% of incidents they held the injured body part. Following 78.2% of the incidents, children did not engage in any type of social response (i.e., neutral); however, in 18.8% of incidents, children engaged in some form of help-seeking behavior (e.g., looking, moving, or making a verbalization toward the parent) and in 3.0% they withdrew from the current play activity. One minute following the everyday pain incident, 94.1% of children’s facial expressions of distress were rated as 0/10 on the FPS-R, 3.0% were scored as 2/10, and 1.0% each were scored as 4/10 and 8/10 (1.0% unknown). Intensity of distress was also rated 1 min following the incident and 98.0% were rated as 0, whereas 1.0% each were rated as a 2/5 and 3/5 (no incidents were rated as a 4/5 or 5/5).

Although parents exhibited a behavioral response after most everyday pain incidents, 15.8% of the 101 incidents were unnoticed by the parent and 24.8% of the time, parents were observed to witness the pain incident but not respond. After 54.5% of the pain incidents, parents made a verbal response. Types of verbal responses included checking in with their child (e.g., “are you OK?” or “did that hurt?”; 16.8%), reassuring them (e.g., “you’ll be OK” or “don’t worry”; 5.0%), talking to them about something else (e.g., change of subject/distraction, “where’s your friend?”; 2.0%), telling them to “be more careful” (e.g., or “watch where you’re going”; 4.0%), sympathizing with them (e.g., “that must have hurt”; 3.0%), and verbally encouraging them to do something else (e.g., “go back and play”; 1.0%). A number of parents (20.8%) also said things like “oops” or “oh-oh” (9%), “you’re so tired” (1.0%), or laughed at them (2.0%). None of the parents apologized to their child (e.g., “I’m sorry”), told them to stop crying, got upset with them, scolded them, or threatened them.

Parents made a nonverbal response after 56.4% of the incidents, which included behaviors such as staying closer to their child (18.8%), hugging or kissing them (13.9%), removing them from the play setting (10.9%), rubbing the hurt area better (8.9%), kissing the “boo-boo” (5.0%), distracting them with a toy (1.0%), and distracting them by pointing to something else (2.0%). Other nonverbal responses included parents picking their child up or helping them to get up (19.8%), attempting to hug child with arms outstretched (1.0%), or helping the child to use the play equipment correctly (e.g., helping them climb the ladder; 3.0%).

### Effect of children’s age, sex, and temperament on children’s responses to everyday pain incidents

As previously described, children were grouped into the following temperament categories based on their TTS scores: very easy (17.3%), easy (38.5%), somewhat easy (40.4%), and somewhat difficult (3.8%). In addition, a “difficult temperament composite score” was calculated for all children (range = 10.84–22.21, M = 16.09, SD = 2.39), which was a continuous measure of the relative difficulty of child temperament.

The relationships between children’s age, sex, and difficulty of temperament (using the difficult temperament composite score) and types of everyday pain incidents experienced (e.g., self-inflicted injuries; being hurt by another child, by an adult, or by an object) were examined. Analyses did not reveal differences between boys and girls on types of everyday pain incidents experienced. Correlations with age and difficulty of temperament as well as a chi-square test conducted with child sex were nonsignificant. Although correlations with age and difficulty of temperament were not related to children’s social responses (i.e., withdrawal or help-seeking behaviors), a chi-square test revealed that boys were more likely to not exhibit any protective behaviors (86.4%) than girls (62.5%), who were likely to hold the injured area (7.5%) or reduce their activity (22.5%), χ^2^ (3, *n* = 100) = 9.75, *P* < 0.05.

Finally, children’s age, sex, and difficulty of temperament (using the difficult temperament composite score) were examined as predictors of their immediate distress to everyday pain incidents and overall pain incidents using regression analyses. These models were nonsignificant.

### Parents’ responses to children’s everyday pain incidents

We examined the frequency with which parents responded to or ignored everyday pain incidents when they were witnessed (Table 2). Point-biserial correlations were computed to examine the relationships between age, sex, difficulty of temperament, and whether parents responded or not. Next, we examined the relationships between child age, sex, and difficulty of temperament (using the difficult temperament composite score) and parent responses. First, a series of correlations was conducted with parents’ verbal and nonverbal responses. Although there were no significant relationships with age, sex, and difficulty of temperament and parents’ verbal responses, child age positively correlated with picking up the child (*r* = 0.38, *P* < 0.05). Thus, parents were more likely to pick up older toddlers. Binary logistic regressions revealed that child age, sex, and difficulty of temperament did not predict whether parents responded verbally/nonverbally or not.

## Discussion

This is the first research report to examine responses to everyday pain incidents among toddlers and their parents during a naturalistic play session. Findings revealed that everyday pain incidents occurred with high frequency among toddlers. Overall, 81% of children experienced at least one everyday pain incident, yielding an overall incident rate of 1.02 incidents/child/hour. This is higher than rates found in previous research with children aged 3–7 years (0.34–0.41 incidents/child/h^1,2^) and could be due to toddlers’ less developed motor skills, an active play setting with adventurous equipment, and presence of parents (who might be less consistent about limits/safety and more likely to reinforce everyday pain) versus daycare staff. The everyday pain experiences that were observed were frequent but relatively short-lasting. Overall, observers rated the majority of pain incidents as resulting in little to no pain (94%) and distress (98%) and distress was observed to completely resolve within 1 min. Previous research examining everyday pain in older children similarly found that the majority of pain incidents were of low severity and caused low levels of distress.^1,2^ Nevertheless, these everyday pain experiences could provide salient learning experiences within which young children’s pain responses are shaped.

Findings revealed that after the majority (76.2%) of incidents, there were no protective behaviors observed; however, 11.9% of the time children reduced their activity or favored (6.9%) or held (4%) the injured body part. For the majority (78.2%) of incidents, children did not engage in any type of social response; however, they occasionally (18.8% of incidents) sought help from their parent or withdrew from the current play activity (3% of incidents). Boys were less likely to exhibit protective behaviors (holding/favoring the injured area) than girls, supporting sex differences in pain behaviors at this young age. Parental responses did not differ by child sex; however, age differences were found in that parents were more likely to pick up older toddlers. Although older children are more independent than younger children, which might be expected to evoke less physical responses by parents, this finding may also be due to older children’s more advanced communicative skills. Indeed, older toddlers might be more instrumental in seeking to be picked up and more effective in eliciting that adult response, whereas younger toddlers may be relatively more helpless due to less sophisticated help-seeking behaviors. This might also reflect the fact that older children have had more time to socially learn how to evoke this response from their parents. The lack of associations between child temperament and responses to children’s everyday pain is consistent with previous research showing that temperament was not related to injury frequency among children aged 2–2.5 years^30^ and may only play a small role in 3- to 7-year-old children’s responses to everyday pain events.^1^

This study was the first to examine parental responses to toddlers’ everyday pain experiences and their relationship with individual characteristics (i.e., age, sex, temperament) and responses of the child. In contrast to responses of adult daycare workers to older children,^1,2^ parents were found to respond more frequently to their children’s everyday pain incidents; only a minority of incidents (15.8%) were observed to go unnoticed by parents. This finding could reflect the low child-to-adult ratio. It may also suggest that parents are more vigilant than daycare workers to children’s everyday pain or that daycare workers may be trained to not react to these minor pain incidents. When parents responded to their children’s everyday pain, which occurred nearly 75% of the time, it frequently (i.e., following 56.4% of the incidents) involved nonverbal responses, such as staying closer to the child, physically picking the child up, hugging or kissing the child, removing the child from the play setting, rubbing the hurt area, kissing the “boo-boo,” or distracting them. It is likely that parents’ own cognitions and learning histories around pain influence whether and how they respond to these pain incidents in their child. More broadly, it is also likely that factors related to parents’ more general tendency to perceive threat in their environments (e.g., trait anxiety) would play a role in the nature of their response to children’s pain. Moreover, parents exhibited verbal responses, which most frequently included checking in with their child (e.g., saying “are you okay?”; “did that hurt”?). Age differences were found; parents were more likely to physically pick up older toddlers. It is likely that parent responses not only serve to reinforce pain behaviors of the child but that individual differences of the child (e.g., age) serve to shape parental responses over time.

This study had limitations and highlights avenues for future research. Firstly, interrater reliability using the DEPS-R was generally good to excellent; however, it was less than adequate for a small number of variables (i.e., activity level, tone, body location, intensity of anger and distress post incident), which were excluded from analyses. We acknowledge that these situational distress behaviors may have a greater impact on observed parent behaviors in the moment than more stable child factors such as sex. This should be examined in future research. The lack of reliability of these particular items on the DEPS-R is consistent with previous research^2^ and suggests that these particular items of the DEPS-R may lack utility and the measure may warrant further revision. The low agreement on some items could be due to a number of factors, including insufficient training, the high ratio of children to observers, or the inclusion of too many items on the DEPS-R. Because observers were assigned to areas and not to individual children and parents, they were frequently not observing the same child at the same time. To reduce bias and increase agreement, future research could use time sampling (i.e., two observers watch the same child for set intervals) and/or limit particular observers exclusively to child or parent behaviors. In addition, it is a limitation that the video quality was of poor quality, which precluded their use for coding and calculation of reliability. To maximize the ability to code from video and to increase reliability, future research could consider increasing the number of coders and reducing the number of dyads to observe. Moreover, other smaller settings such as home environments and daycares might be more amenable to use of the DEPS-R in this way. In addition, we did not collect data on the actual amount of time the children were playing but noted that they played for the vast majority of the time, with the exception of when they were having a snack (a snack table was available to all participants). The stated expectation of participants at the time of study enrollment was for them to stay for the entirety of the play party and very few families were observed to leave early; however, it is a limitation that this information was not systematically recorded.

Future psychometric examinations are needed to establish the validity of the DEPS-R. Of note, facial expression of child distress, as assessed using the Faces Pain Scale–Revised, was more reliable than intensity of distress measured using a numerical rating scale. Combined with previous findings,^2^ this indicates that in this context, facial expression may be a particularly salient cue for observers to recognize child distress and reliably translate this judgment using a scale. Nevertheless, observational scores on the FPS-R to assess facial expression and intensity of distress 1-min post pain incident had limited variability. We acknowledge that the FPS-R was designed to capture self-report of pain. We note that previous studies have used faces pain scales by adults to measure children’s pain,^31,32^ which generally show moderate agreement with child self-report. However, given that faces scales have not been well studied as observational tools, they have limited evidence for validity and interpretability when used in this manner. It is also possible that observer bias could have played a role in this study. For example, all of the coders were female, which might have influenced ratings (e.g., differences between ratings of protective responses for boys versus girls). Future studies should strive to utilize methods to minimize sources of bias (e.g., ensuring equal representation of gender and cultural background of raters). In addition, the sample was homogeneous due to a low number of fathers and mostly Euro-Canadian participants of high-socioeconomic status background, thereby limiting the generalizability of the findings. Indeed, parent responses to young children’s everyday pain may be influenced by sociocultural factors, and this warrants further research. Although the exact duration of child distress was not recorded in the present study, Fearon and colleagues^1^ reported a very short mean duration of distress in older children (i.e., average of 7.9 s). As such, future research should obtain observational ratings sooner in order to capture greater variability in post pain incident distress. Future research should also examine sequential relationships^33^ between child and parent responses in order to capture shaping and reinforcement processes over time. Finally, we argue that one of the most important areas for future research is to examine the relationship between parent responses to everyday pain and their responses to clinical acute pain experiences (e.g., medical procedures). We hypothesize that responses to everyday pain may be influenced by early clinical pain experiences (e.g., infant immunizations) but might also socialize pain behaviors that influence how pain is experienced in future clinical settings. This has not yet been empirically examined but has important implications for prevention.

Bumps and scrapes are common causes of pain in children and although they rarely result in serious injuries, the high frequency with which they occur creates valuable opportunities for children to learn about pain from adults. We argue that the parent–child interactions occurring within this context likely reflect dynamics and response styles that are typically exhibited in children’s everyday lives. Thus, this context offers a rich and ecologically valid milieu in which to study pain learning processes during a sensitive developmental period, when parents’ influence on children’s development is strongest. Several of the responses captured in the current study are consistent with those assessed in older children that have been linked to increases (reassurance) and decreases (distraction) in child pain and distress^34^; however, the function and influence of these responses across various stages of childhood is unclear. The current findings can be used to stimulate pain research during the toddler period so that parenting processes in the context of child pain can be readily examined across infancy, childhood, and adolescence. Future research should examine the link between parents’ responses in everyday and clinical pain contexts and determine whether this period offers a window for parent intervention to improve children’s pain trajectories.

## Appendix B: Operational Definitions for DEPS-R

**Behavioral Context**

- Activity Level (1–5). How much activity is the child displaying before the hurt takes place?
  - E.g., Sitting = 1, Running = 5
- Tone (1–5). What is the temper or mood of the child prior to the hurt?
  - E.g., Calm = 1, Excited/Upset = 3, Agitated = 5

**Description of Incident**

- Body Location. Where on the body is the child hurt?
  - Circle all that apply
- Hurt Caused By. Who or what caused the child’s hurt?
  - Self: falling, slipping, running, bumping
  - Other child: getting hit by, toy thrown at
  - Adult: spanking, pushing, “letting go of child”
  - Object: trips on ladder, falls off slide
- Severity of Hurt (0–4). How much pain, as opposed to emotional upset, do you believe you would experience in the subject’s place?
  - E.g., child lightly falls over on mat = 0, child falls off jungle gym = 4

**Subject’s Response**

- Intensity of Distress *Before/After*Parent Intervention (0–5). How much distress does the child show as a direct reaction to the pain?
  - 0: None
  - 1: Some facial or verbal expression of pain (e.g., “ouch!”)
  - 2: Greater facial or verbal expression of pain (e.g., obvious facial distress)
  - 3: Sobbing/whimpering
  - 4: Crying
  - 5: Screaming
- Facial Expression of Distress *Before/After*Parent Intervention (FPS). Which face looks closest to the face of the child experiencing the pain?
- Intensity of Anger (0–5). How much anger does the child show following the pain incident?
  - E.g., No anger = 0, throwing a toy angrily = 3, child hits other child/parent or having a temper tantrum = 5
- Protective Behaviors. What type of behavior directed at the hurt does the child engage in following the incident?
  - Holding: hand on top of hurt
  - Favoring: rubbing, holding up to face, looking at hurt
  - Reduction of activity: if child plays less actively within one minute of the incident
- Social Response. What does child do socially following pain incident?
  - Withdrawal: Removes self from current play activity
  - Neutral: Remains in present state
  - Help-seeking: Looks to parent, looks to peer, moves toward parent, verbalizations toward parent etc.

**Parent Response Checklist**

- ***Verbal Behaviors.***
  1. Reassures child that s/he will “be OK”
    - “It’s OK”; “you’re OK”; “It’s alright”; “Don’t worry”; “It will be OK”; “Everything will be alright”
  2. Apologizes to child
    - “I’m sorry”
  3. Sympathizes with child (e.g., tells them they know how much it hurts)
    - “I know it’s sore”; “That must have hurt”
  4. Encourages child to do something else
    - “Go get a toy”; “Go back and play”
  5. Talks about something else. Any change of subject (distraction)
    - “Where is your friend?”; “Look over there”; “What do you want for dinner?”
  6. Tells child to “stop crying”
    - “Don’t cry”; “No more tears”
  7. Tells child that s/he should be more careful
    - “Be careful”; “Watch where you’re going”
  8. Checks in with child
    - “Are you OK?”; “Are you alright?”; “How are you feeling?”; “Did that hurt?”
  9. Gets upset, scolds, or threatens child
    - “If you don’t stop we’re going to leave”; I’ve had enough of you/this”
  10. Other
    - Record any other verbal behaviors you can hear

- ***Nonverbal Behaviors.***

1. Doesn’t notice incident
  - Parent does not see child get hurt and makes no response
2. Witnesses incident but shows no reaction
  - Any evidence that parent saw the child get hurt but makes no apparent reaction/response during 1-minute period
3. Rubs the hurt area better
  - Any attempt to rub, pat, caress the hurt area
4. Hugs or kisses the child
  - Hugging or kissing the child (not “booboo”)
5. Kisses the “booboo” better
  - Any attempt to kiss the hurt area
6. Stays closer to child
  - Stays closer to child than before the incident occurred, monitors child more closely
7. Distracts child with a toy or object
  - Brings child a toy or food or anything to draw his/her attention away from hurt
8. Distracts child by pointing to something
  - Tries to engage child’s attention using a pointing gesture
9. Takes child out of play setting
  - Parent picks up child or other wise removes them from the play setting
10. Other

- Record any other nonverbal behaviours you can see
